# Leveraging long-acting IL-15 agonists for intratumoral delivery and enhanced antimetastatic activity

**DOI:** 10.3389/fimmu.2024.1458145

**Published:** 2024-11-04

**Authors:** John A. Hangasky, Rocío del Valle Fernández, Dimitris Stellas, Guillermo Hails, Sevasti Karaliota, Gary W. Ashley, Barbara K. Felber, George N. Pavlakis, Daniel V. Santi

**Affiliations:** ^1^ ProLynx Inc., San Francisco, CA, United States; ^2^ Human Retrovirus Pathogenesis Section, Vaccine Branch, Center for Cancer Research, National Cancer Institute at Frederick, Frederick, MD, United States; ^3^ Basic Science Program, Frederick National Laboratory for Cancer Research, Leidos Biomedical Research, Inc., Frederick, MD, United States; ^4^ Center for Cancer Research, National Cancer Institute at Frederick, Frederick, MD, United States

**Keywords:** interleukin-15, prolonged release formulation, immunotherapy, natural killer cells, intra-tumoral therapy, management of metastases

## Abstract

**Introduction:**

IL-15 agonists hold promise as immunotherapeutics due to their ability to induce the proliferation and expansion of cytotoxic immune cells including natural killer (NK) and CD8^+^ T cells. However, they generally have short half-lives that necessitate frequent administration to achieve efficacy. To address this limitation, we have developed a half-life extension technology using hydrogel microspheres (MS). Here, the therapeutic is tethered to MSs by a releasable linker with pre-programed cleavage rates. We previously showed the MS conjugate of single-chain IL-15, MS~IL-15, effectively increased the half-life of IL-15 to approximately 1 week and enhanced the pharmacodynamics. We sought to determine whether the same would be true with a MS conjugate of the IL-15 agonist, receptor-linker IL-15 (RLI).

**Methods:**

We prepared a long acting MS conjugate of RLI, MS~RLI. The pharmacokinetics and pharmacodynamics of MS~RLI were measured in C57BL/6J mice and compared to MS~IL-15. The antitumor efficacy of MS~RLI was measured when delivered subcutaneously or intratumorally in the CT26 tumor model and intratumorally in the orthotopic EO771 tumor model.

**Results:**

MS~RLI exhibited a half-life of 30 h, longer than most IL-15 agonists but shorter than MS~IL-15. The shorter than expected half-life of MS~RLI was shown to be due to target-mediated-disposition caused by an IL-15 induced cytokine sink. MS~RLI resulted in very potent stimulation of NK and CD44^hi^CD8^+^ T cells, but also caused significant injection-site toxicity that may preclude subcutaneous administration. We thus pivoted our efforts toward studying the MS~RLI for long-acting intra-tumoral therapy, where some degree of necrosis might be beneficial. When delivered intra- tumorally, both MS~IL-15 and MS~RLI had modest anti-tumor efficacy, but high anti- metastatic activity.

**Conclusion:**

Intra-tumoral MS~RLI and MS~RLI combined with systemic treatment with other agents could provide beneficial antitumor and anti-metastatic effects without the toxic effects of systemic IL-15 agonists. Our findings demonstrate that intra-tumorally administered long-acting IL-15 agonists counter two criticisms of loco-regional therapy: the necessity for frequent injections and the challenge of managing metastases.

## Introduction

Interleukin-15 (IL-15) has emerged as a promising immunotherapeutic agent for the treatment of cancer, and other immune-related disorders (1–4). IL-15 plays a crucial role in facilitating the survival, differentiation, as well as activation and expansion of Natural killer (NK) cells, cytotoxic CD8^+^ T cells, and γδ T cells without expanding immunosuppressive regulatory T cells (T_regs_) or mediating activation-induced cell death (5, 6). As such, IL-15 is a popular and attractive candidate for immunotherapy.

**Figure 1:**
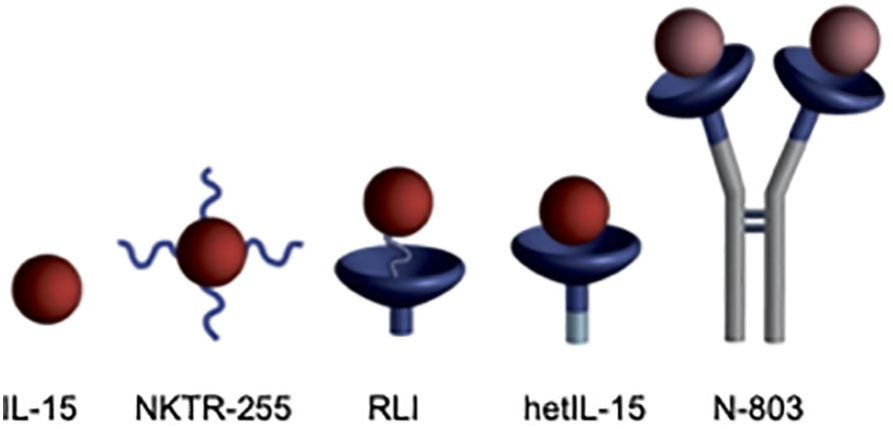
IL-15 agonists. i) IL-15, ii) NKTR-255 is PEGylated IL-15 (17), (iii) RLI has a 20 amino acid linker connecting C-terminus of the IL-15Rα Sushi domain to the N-terminus of IL-15, (iv) hetIL-15 is the non-covalent heterodimeric IL-15/IL-15Rα complex similar to the endogenous cytokine, and (v) N-803 is an Fc-IL-15Rα sushi domain/IL-15N72D fusion. Figure reproduced with permission from Ref (28).

The biologically active form of IL-15 in the body is the non-covalent heterodimeric IL-15/IL-15Rα complex (7). The single chain IL-15 polypeptide is not found in nature, because it is coordinately expressed with and stably bound to the so-called “IL-15Rα“. Numerous IL-15 agonists, consisting of the soluble IL-15Rα domain covalently or non-covalently fused to IL-15, have been reported to have superior pharmacokinetic (PK) and biological activity than the single chain IL-15 polypeptide (8–11). Several IL-15 agonists have shown promising results in preclinical and early-phase clinical trials (**Figure 1**), and one, N-803 (Anktiva), has recently been approved for treatment of BCG-unresponsive non muscle invasive bladder cancer. However, a prevalent issue among all IL-15 agonists is their short apparent half-lives. For instance, receptor-linker IL-15 (RLI) (12, 13), the heterodimeric IL-15/IL-15Rα complex, hetIL-15 (10, 14), and N-803 (15, 16) have apparent half-lives of 8 h or less in mice, ~12 h in nonhuman primates (NHP) and up to 30 h in humans. Therefore, these IL-15 agonists require frequent and inconvenient dosing schedules.

**Figure 2:**
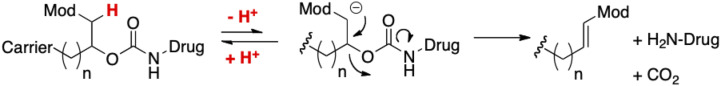
General approach to half-life extension. A β-elimination reaction slowly releases a covalently attached drug from a long-lived carrier.

We have developed a general approach to increase the residence time of drugs through the covalent attachment of a releasable linker to non-circulating ~50 µm diameter tetra-PEG hydrogel microspheres (MS) (18–21). Drug release occurs via a β-elimination reaction, and the release rate is controlled by a rate modulating (Mod) electron-withdrawing group (**Figure 2**). MS conjugates can be injected subcutaneously (SC) or intratumorally (IT) through a 29G needle to serve as a localized drug depot that can sustain drug release for weeks to months (22, 23). In addition, β-eliminative linkers are engineered into the polymers of the MS hydrogels to enable gel dissolution subsequent to drug release (24).

We previously reported a MS hydrogel conjugate of IL-15 with a long apparent half-life (t_1/2_) (25). The MS depot provided continuous release of IL-15 with an initial half-life of ~5 days, and improved PK, pharmacodynamics (PD) and efficacy. We sought to determine if an analogous MS conjugate of the IL-15 agonist RLI (11) could also extend the apparent half-life and improve PD over the free drug. Herein, we present the synthesis, *in vitro* and *in vivo* characterization, and antitumor efficacy of a MS~RLI prodrug. We describe the effects of MS~RLI on its target immune cells when administered SC, and the anti-tumor and anti-metastatic effects when administered IT. We propose that the sustained release facilitated by the conjugation to the MSs is an attractive approach for loco-regional delivery of IL-15 agonists. The prolonged IT release of an MS~IL-15 agonist in combination with a second agent, administered systemically or locally, should improve the antitumor and anti-metastatic effects minimizing systemic toxic effects of the IL-15 agonist.

## Materials and methods

Complete, detailed descriptions and procedures used for the synthesis and characterization of MS~RLI, PK studies and PD studies are provided in the **Supplemental Information**.

### Materials

RLI (>95% pure) was produced at ATUM (Newark, CA) based on previously reported methods (11). MS~IL-15 was prepared according to published procedures (25). All other reagents were purchased from commercial vendors and used as received.

### Synthesis of MS~RLI

Reductive alkylation using NaCNBH_3_ was used to conjugate N_3_-PEG_4_-linker(MeSO_2_)-CHO to RLI. The excess reagents were removed using prepacked PD-10 columns (GE Healthcare). The azido-linker-RLI was attached to bicyclononyne(BCN)-derivatized MSs via strain promoted azide-alkyne cycloaddition (SPAAC). Then, N_3_-PEG_7_ (Sigma Aldrich) was used to cap the unreacted BCN groups. Finally, the MS~RLI conjugate was extensively washed and equilibrated in 25 mM Citrate, 500 mM NaCl, 30 mM methionine and 0.05% tween-20, pH 6.0 and stored at 4°C.

### Cell-based assay

A U2OS cell-based assay kit for IL-2/IL-15Rβγ binding was performed according to the manufacturer’s (DiscoverX, Part #93-0998E3CP5).

### In vivo PK and PD studies

For PK studies, MS~RLI was administered SC to either male C57BL/6J mice or male NSG mice. Alternating groups of animals were bled and blood samples were collected in EDTA collection tubes containing HALT protease inhibitor. Plasma was prepared and stored at –80°C until analysis. Plasma samples were thawed on ice prior to analysis by either a hIL-15/IL-15Rα complex specific ELISA (R&D Systems, hIL-15/IL-15Rα complex DuoSet ELISA, Catalog #DY6924) or ELLA protein simple kit (Catalog SPCKB-PS-000500). For PD studies, MS~RLI was administered SC to male C57BL/6J mice. Mice were bled over a 28 day period. EDTA whole blood was collected and immunophenotyped by flow cytometry.

### CT26 syngeneic model

CT26 tumors were established in the flank of female BALB/c mice by injection of 1 x10^5^ in 100 µL of serum-free medium. When the tumor volume reached ~100mm^3^ mice were randomized into groups (n=7-8/group) and mice were administered empty MSs IT, 10 µg MS~RLI IT, or empty MSs IT plus 10 µg MS~RLI SC. The experiment was performed two times. In the second experiment, mice (n=4/group) were sacrificed on day 5 and EDTA whole blood, tumors and spleens were harvested for immunophenotyping. Kaplan-Meier mouse survival plots were generated based on the mouse survival, monitored based on humane end point criteria. The tumor volume was measured by calipers and calculated by the equation: V = 1/2(long dimension)(short dimension)^2^.

### EO771 orthotopic model of Triple Negative Breast Cancer (TNBC)

EO771 tumors were established by orthotopic injection of 3x10^5^ EO771 cells into the 4th mammary pad of C57BL/6 mice. A single 50 µL IT dose of MS~IL-15 (1.2 μg or 6 μg) or MS~RLI (2 μg or 10 μg) was administered when the tumors reached ~40 mm^3^; empty MS (50 µL) were used as the negative control. Blood was collected 6 days before treatment, one day after MS administration and at the end of the experiment. Tumor volume was measured by calipers and calculated by the following equation: L*W*H*p/6. All mice were sacrificed on day 15 of the treatment. Tumor-infiltrating immune cells and splenocytes were analyzed by flow cytometry. Lungs were embedded in paraffin and the metastatic lesions were evaluated by hematoxylin/eosin staining.

### Flow cytometry

For PD studies in naïve mice, EDTA whole blood was incubated with a fixable viability dye, followed by incubation with CD16/32 antibody and subsequently surface stained with previously determined optimized Ab concentrations (**Supplementary Table 1**). Red blood cells (RBCs) were lysed and peripheral blood mononuclear cells (PBMCs) were fixed using a 1-Step Fix/Lyse solution (Invitrogen). A 1x permeabilization buffer (Invitrogen) was used to permeabilize PBMCs for intracellular staining.

For immunophenotyping of tumor bearing animals, EDTA whole blood was stained as described above, but RBCs were lysed with 1x RBC lysis buffer (Invitrogen) and for intracellular staining, cells were fixed and permeabilized using the Foxp3 Transcription Factor Staining Buffer Set (Invitrogen). Splenocytes were obtained from harvested spleens following mechanical disruption and filtering through a 40-µm cell strainer. RBCs were lysed using 1x RBC Lysis Buffer. Tumor infiltrating lymphocytes (TILs) were obtained from excised tumors by mechanical and enzymatic digestion and the lymphocytes were purified using Lympholyte-Mammal Cell Separation Media gradient (Cedarlane). Single cell suspensions (1x10^6^ cells) were stained with previously determined optimized Ab concentrations (**Supplementary Table 1**). Cells were fixed and stained intracellularly using the Foxp3 Transcription Factor Staining Buffer Set.

Stained single cell suspensions were read using a Attune NxT flow cytometer (BD Biosciences) and analyzed using FlowJo cytometry analysis software (TreeStar, Ashland, OR). A representative gating strategy is shown in **Supplementary Figure 1**. CD8^+^ and memory CD8^+^ T cells were identified as CD3^+^CD8^+^ and CD3^+^CD8^+^CD44^hi^, respectively; NK cells were identified as CD3^-^NK1.1^+^ or CD3^-^CD49b^+^NKp46^+^. Treg cells were identified as CD3^+^CD4^+^CD25^+^Foxp3^+^. Cells with proliferative capacity were defined as Ki67^+^.

### Statistics

The statistical testing method used is reported in each figure legend. One-way ANOVA followed by Tukey’s multiple comparison test were used for analysis of immunophenotyping data. Tumor volumes were plotted as mean ± standard error of the mean and compared using two-way ANOVA or mixed-effects model. Kaplan-Meier survival was analyzed by Mantel-Cox. All statistical testing was performed using GraphPad Prism v.9.5.1. A *p* value of less than or equal to 0.05 was considered statistically significant in all analyses.

## Results

### Preparation and in vitro characterization of MS~RLI

**Figure 3:**
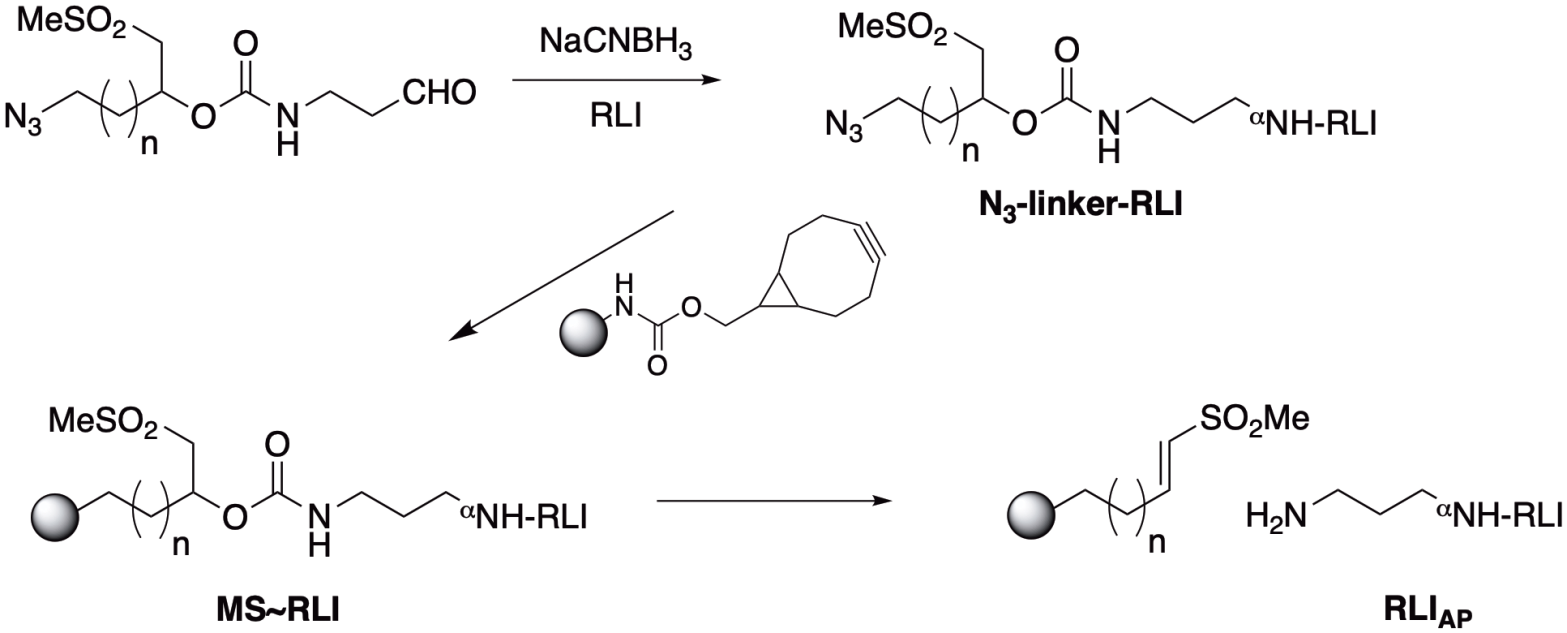
Synthesis of MS~RLI and RLI_AP_ release. Note that upon β-elimination the aminopropyl moiety of the linker is transferred to the protein to give aminopropyl-RLI (RLI_AP_) as the final product.

We first sought to prepare a long-acting RLI by attaching the agonist to hydrogel MS via a β-eliminative releasable linker. To preclude spontaneous deamidation at N77 we introduced the N77A substitution into the protein sequence (26). Then, we prepared the MS~RLI prodrug following the successful strategy used for the analogous MS~IL-15 (**Figure 3**) (25).

First, site-specific reductive alkylation of RLI using NaCNBH_3_ was used to attach an azido-linker carbamate of aminopropyl aldehyde to the N-terminus (27). Small scale reactions showed that a linker/RLI of 2:1 was optimal to give the highest balanced yield of monoalkylated N_3_-linker-RLI (**Supplementary Figure 3**). Then, 18 mg of RLI was treated with 2 equivalents of N_3_-linker-aldehyde and 10 mM NaCNBH_3_ at pH 6.0. A PEG-shift SDS-PAGE assay (22) showed ~45% unreacted RLI, ~55% RLI having one PEG and ≤5% with more than one PEG; thus, ≥90% of the alkylated product was the desired monoalkylated RLI. Without further purification, the N_3_-linker-RLI was coupled to BCN-derivatized tetra-PEG MS by SPAAC, and unreacted BCN groups were capped with N_3_-PEG_7_. The MS were extensively washed to remove unbound RLI and exchanged into pH 6.0 buffer containing 30 mM Met, and stored at 4°C. The packed slurry of 1 mL contained 4.2 mg/mL RLI (184 nmol) and had a PEG content of 3.5 mg/mL. Thus, the packed MS slurry contained 52 nmol RLI/mg PEG indicating the MS were loaded to 52%.

We characterized MS~RLI *in vitro* to ensure the release rate was suitable and that the released aminopropyl-RLI (RLI_AP_) was biologically active. At pH 9.4, the base-catalyzed β-elimination of the MS~RLI resulted in complete release of RLI_AP_, with a t_1/2,pH9.4_ of 7 h corresponding to 700 h at pH 7.4 (**Supplementary Figure 4**). The MS gel contained GDM DMS modulators in the crosslinks, and the *in vitro* time to reverse gelation (t_RG_) at pH 9.4 was 28 h, corresponding to 2,800 h at pH 7.4 (**Supplementary Figure 4**). The purity of RLI on the MS~RLI assessed by HPLC quantitation of released protein vs time at pH 9.4 was ≥93% (25) (**Supplementary Figure 5**). Finally, using an engineered U2OS cell line expressing the human IL-2/IL-15Rβ/γ_c_ the RLI_AP_ released from the MS at pH 7.4 retained an EC_50_ equipotent to native RLI, demonstrating that coupling to and the release of RLI from MS does not affect receptor engagement (**Supplementary Figure 6**). These results showed that MS~RLI slowly releases RLI_AP_ with a long *in vitro* half-life, that the released RLI_AP_ retained full bioactivity, and that the MS~RLI was completely suitable for *in vivo* studies.

### MS~RLI increases the apparent half-life and exposure of RLI_AP_

**Figure 4:**
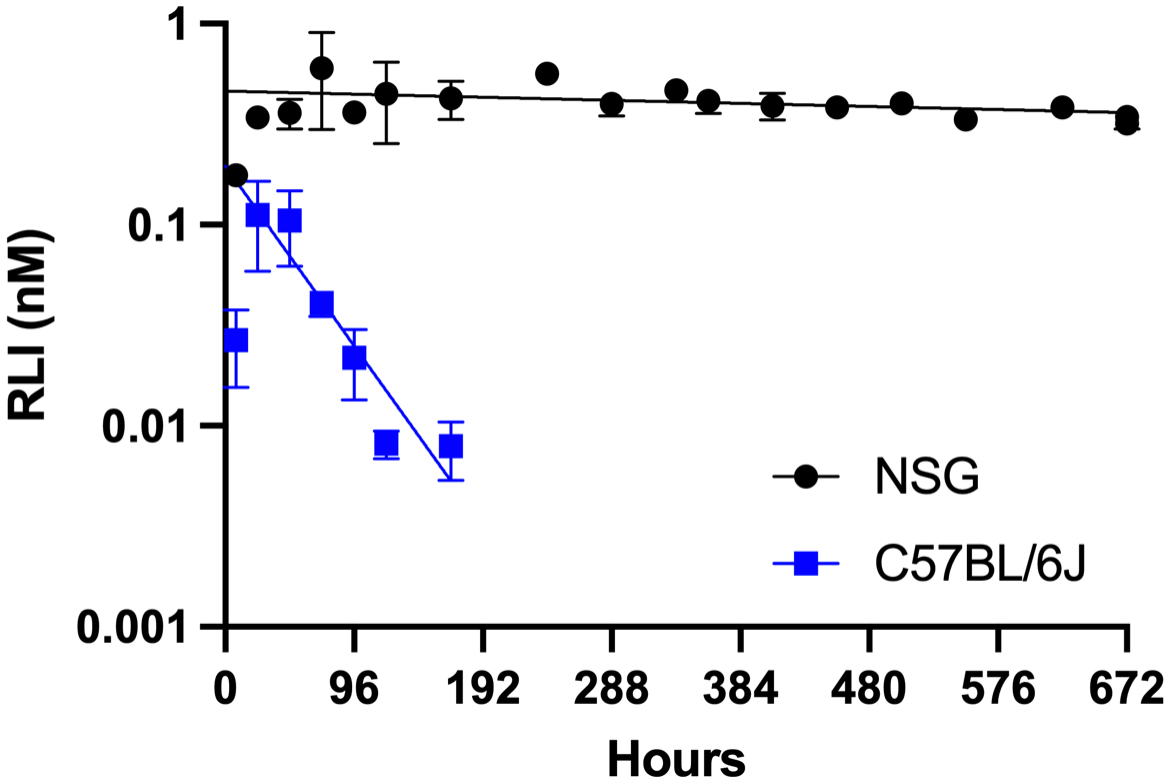
Log-linear plasma C vs t plot of RLI_AP_ released from MS~RLI in C57BL/6J and NSG mice. NSG mice (●) and C57BL/6J mice (■) were administered MS~RLI_10µg_ on Day 0 and RLI_AP_ plasma concentrations were determined by ELISA. Data represented as mean ± SD.

To evaluate the PK properties of MS~RLI in mice, plasma concentrations of RLI_AP_ were determined after a single SC dose (**Figure 4**). The C vs t plot of MS~RLI_10µg_ in C57BL/6J immunocompetent mice showed a terminal half-life of ~30 h for the released RLI_AP_. This is a 10-fold increase in half-life over free RLI (3 h), but much lower than expected from the 700 h half-life for *in vitro* cleavage; in our experience, MS~drug conjugates show *in vivo* half-lives of release that are ~2- to 3-fold lower than the *in vitro* release (19). Moreover, although the MS~RLI conjugate uses the same linker (Mod = MeSO_2_-) as MS~IL-15, it shows a much faster rate of release (t_1/2_ 30 vs 168 h). We hypothesized that, as proposed for other IL-15s, the higher than expected clearance of RLI might be due to the rapid formation of an immune cell cytokine sink (28). Therefore, the PK of MS~RLI_10µg_ was determined in NSG mice lacking IL-2/IL-15R**γ**c and deficient in NK, B and T cells. The C vs t plot of the RLI_AP_ released from MS~RLI_10µg_ in NSG mice showed sustained plasma concentrations for at least one month with a remarkably long half-life of ~700 h (**Figure 4**). This >20-fold increase of t_1/2_ in immunodeficient mice is consistent with presence of an immune-cell cytokine sink at the onset, which may increase by the generation of additional lymphocyte targets. Summarizing, the above results show that a) the *in vivo* apparent half-life of RLI_AP_ was extended 10-fold compared to RLI, b) the observed half-life was much shorter than expected from the same linker used with MS~IL-15, and c) the short half-life of released RLI_AP_ is due to an immune cell cytokine sink.

We next sought to construct a PK model that supports the PK data. The standard equation describing the plasma concentration of a drug released from a depot in a first-order process with rate constant k_1_ and subsequently eliminated from the plasma in a first-order process with rate constant k_2_ (Equation 1) was modified to include a time-dependent elimination rate constant (Equation 2) wherein a basal non-target mediated elimination rate, k_basal_, (renal filtration, for example) is augmented by an immune cell-mediated elimination rate, k_IC_, that is a function of the number of immune cells present. It is assumed that upon stimulation by the released cytokine the immune cells initially present (N_0_) begin expanding with a doubling time T_d_, such that the rate of elimination of the cytokine from the plasma increases with time.


(1) $$\text{C}\left(\text{t}\right)=\text{Dose}•\text{F}/{\text{V}}_{\text{d}}•{\text{k}}_{1}/\left({\text{k}}_{2}–{\text{k}}_{1}\right)•\left({\text{e}}^{-\text{k}1\text{t}}–{\text{e}}^{-\text{k}2\text{t}}\right)$$

(2) $${\text{k}}_{2}\left(\text{t}\right)={\text{k}}_{\text{basal}}+{\text{N}}_{0}•{\text{k}}_{\text{IC}}•\text{Σ}\left(2^{\text{t}/\text{Td}}\right)$$

For mixed immune cell populations having different doubling times, for example NK cells and T cells, the total cytokine sink is taken as the sum of the different cell populations each with its own T_d_. The simple form of the model assumes that each immune cell type is equivalent in its ability to clear cytokine.

As NSG mice are NK and T cell-deficient (N_0_ = 0), the value of k_2_ that best fits the data from NSG mice is taken as k_basal_. This then allows a determination of the cytokine release rate (t_1/2_ = 700 h) and basal plasma clearance (0.75 mL/h) from fitting the data to a dose of 10 µg (0.44 nmol). Basal plasma clearance was parsed into V_d_/F = 21.6 mL and t_1/2(basal)_ = 20 h based on the observed T_max_ = 72 h. This V_d_/F is in good agreement with the value of 26 mL reported for intraperitoneal administration of RLI in C57BL/6J mice (13). Thus, the high release rate of MS~RLI in immune-competent mice could be closely simulated by a target-mediated drug disposition model (TMDD), where the amount of target increases exponentially over time due to RLI-stimulated NK and T cell expansion – a “cytokine sink”.

### MS~RLI induces high and prolonged CD8^+^ and NK cell expansion in naïve mice

We next determined the effects of MS~RLI on the target immune cells – NK, CD8^+^ and CD44^hi^CD8^+^ T cells –comparing them to those of MS~IL-15_50µg_. We studied the effects of 1- to 10 µg MS~RLI on the expansion of immune cells compared to the optimal 50 µg dose of MS~IL-15 (**Supplementary Figure 7**). The effects at the lower doses, 1-to 2.5 µg, were near-indistinguishable from control. The highest 10 µg dose (~8.8-fold lower equivalents than MS~IL-15_50µg_) more effectively expanded NK cells but showed comparable expansion of CD44^hi^CD8^+^ T cells. Although we did not observe a saturating target immune cell response and 10 µg MS~RLI was well tolerated (**Supplementary Figure 8**), higher doses resulted in injection site toxicities preventing longitudinal PD measurements (vide infra). Thus, we chose MS~RLI_10µg_ as a standard dose for subsequent studies to avoid toxicities and synchronize effects with MS~IL-15_50µg_ on CD44^hi^CD8^+^ T cells.

**Figure 5:**
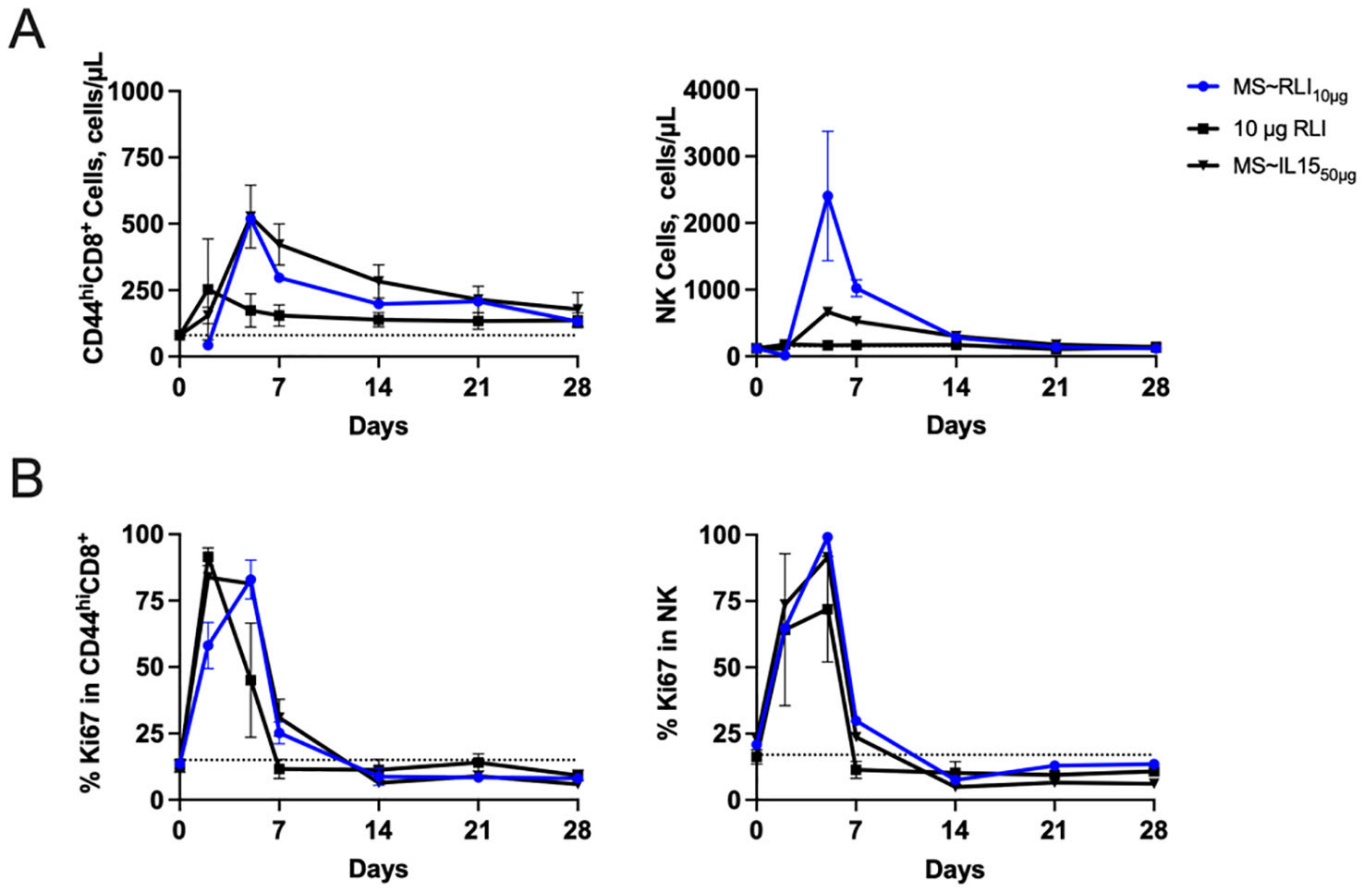
MS~RLI_10µg_ treatment increases the number of CD44^hi^CD8^+^ T cells and NK cells compared to free RLI. **(A)** Absolute cell number and **(B)** Percentage of proliferating CD44^hi^CD8^+^, and NK cells. Mice (n=5/group) were administered a single IP dose RLI (10 µg), a single SC dose of MS~IL-15_50µg_ or MS~RLI_10µg_. Data represented as mean ± SD (n=5/group).

Next, we quantitated the effects of MS~RLI_10µg_ on the target immune cells and compared them to those of MS~IL-15_50µg_ and a bolus injection of free RLI. **Figures 5A, B** shows the longitudinal immune cell expansion, and **Table 1** quantitates the expansion over time by the area under the curve (AUC) method previously reported (25). First, we compared MS~RLI_10µg_ to 10 µg free RLI. MS~RLI_10µg_ caused the same or slightly longer duration of expansion of target immune cells, a ~2-fold increase in the CD44^hi^CD8^+^ T cell AUC_28d_, a high 10-fold expansion in the NK cell AUC_28d_, and a ~12-fold increase in the Ki67^+^ NK cell AUC_28d_. Next, we compared MS~RLI_10µg_ to the long-acting MS~IL-15_50µg_. MS~RLI_10µg_ had a similar expansion of CD44^hi^CD8^+^ T cells compared to MS~IL-15_50µg_, but a 2-fold higher NK cell AUC_28d_, and a 3-fold higher AUC_28d_ for Ki67^+^ NK cells. Finally, a second dose of MS~RLI at day 35 failed to induce proliferation of both NK and CD44^hi^CD8^+^ T cells; other agonists predominantly cause only NK hypo-responsiveness (**Supplementary Figure 9**) (29). Therefore, long-acting MS~IL-15 and MS~RLI both exhibit potent and prolonged immunostimulatory effects on NK cells and CD44^hi^CD8^+^ T cells. However, achieving these PD responses requires lower doses of MS~RLI.

**Table 1:** Duration of immune cell expansion and AUC_28d_ in PBMCs.

Cell Marker	Duration, days			AUC, cells/μl *10^-3^ *d		
	10 µg RLI	50 µg MS~IL-15	10 µg MS~RLI	10 µg RLI	50 µg MS~IL-15	10 µg MS~RLI
CD3^–^NK1.1^+^	14	14	14	1.0	5.5	11
CD8^+^	21	21	21	4.3	4.3	6.8
CD44^hi^CD8^+^	28	28	28	2.0	5.3	4.5
Ki67^+^ CD3^–^NK1.1^+^	7	14	14	0.61	2.1	7.3
Ki67^+^ CD8^+^	14	14	14	1.2	3.0	3.5
Ki67^+^CD44^hi^CD8^+^	7	14	14	0.65	1.7	2.3

### Local toxicity of MS~RLI

The injection site toxicity of MS~RLI was assessed in both immunocompetent and immunodeficient mice. When dosed SC in immunocompetent C57BL/6J mice, at ≥ 20 µg MS~RLI resulted in ≥90% (26/30) of mice forming observable injection site lesions lasting 7- to 14 days post dose administration. We suspected that high local concentrations of RLI was inducing a strong local immune cell activation at the injection site. Indeed, the 10 µg dose of MS~RLI used here for PD studies showed only 2.5% (1/40) of mice showing injection site toxicity; no lesions (0/10) were observed with an equimolar bolus dose of RLI which does not remain at the injection site. H&E staining of the injection site in mice receiving ≥20 µg MS~RLI revealed a range of histologic findings including mononuclear inflammation, epidermal hyperplasia and transmural coagulative necrosis of the dermis, subcutis and brown adipose tissue. There were no observable injection site lesions in immunodeficient NSG mice (n=0/10) at 30 µg MS~RLI, indicating the observed lesions result from the high, prolong exposure from MS~RLI. Thus, while 10 µg MS~RLI appears safe and effective, ≥20 µg MSI~RLI causes severe injection site reactions consequent to immune cell activation by the slow-releasing depot – a therapeutic index of only ~2.

### Intra-tumoral administration of MS~RLI and MS~IL-15 have anti-metastatic activity

**Figure 6:**
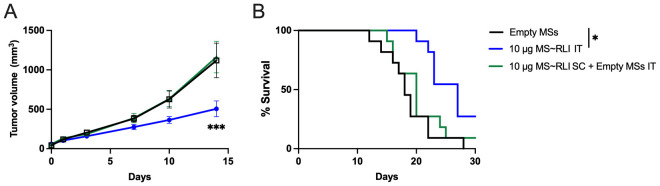
Anti-tumor effect of MS~RLI in CT26 tumor bearing mice. **(A)** Mean tumor volume vs. time post treatment; mice were administered empty MSs IT, 10 µg MS~RLI IT, or empty MSs IT plus 10 µg MS~RLI SC. Data is pooled from 2 independent experiments (n=11/group). **(B)** Kaplan Meier plot of overall animal survival. The median survival times for the control (18 d), SC MS~RLI (20 d) and IT MS~RLI (27 d) are based on the tumor volume reaching 2,000 mm^3^ or animal death. Tumor growth volumes were analyzed by two-way ANOVA, ***p<0.001. Kaplan Meier plot was analyzed by Mantel-Cox test, *p<0.05.

In view of the skin necrosis and consequent low therapeutic index for SC MS~RLI, we studied the effects of IT administration. Since the skin toxicity limited the SC dose to 10 µg MS~RLI, we focused on the same dose IT. Initially, we examined the effects of IT MS~RLI on both CT26 colon carcinoma and EO771 TNBC to assess which was most appropriate for IT studies; the effects of systemic RLI on CT26 (30) and peritumoral hetIL-15 on EO771 (31) have been reported. CT26 tumor bearing mice well tolerated both SC and IT 10 µg MS~RLI with no signs of body weight loss (**Supplementary Figure 10**). However, SC MS~RLI had no effect on tumor growth, whereas IT administration resulted in 40% tumor growth inhibition (TGI) and increase in survival (**Figure 6**). We also observed that both SC and IT MS~RLI caused a ~2-fold increase in the total number of NK cells and high levels of proliferating NK cells in the tumor, blood and spleen (**Supplementary Figure 11**); here, the systemic effect of the high 10 μg IT MS~RLI is likely a manifestation of exposure after released RLI_AP_ exited the tumor. In our early studies of EO771 tumors, the same 10 μg dose of IT MS~RLI caused a similar TGI as in CT26 tumors. Since both tumors had similar responses to IT MS~RLI, we chose to focus on EO771 so we could benefit from the information reported from studies of peritumoral administration of the IL-15 agonist hetIL-15 (31).

**Figure 7:**
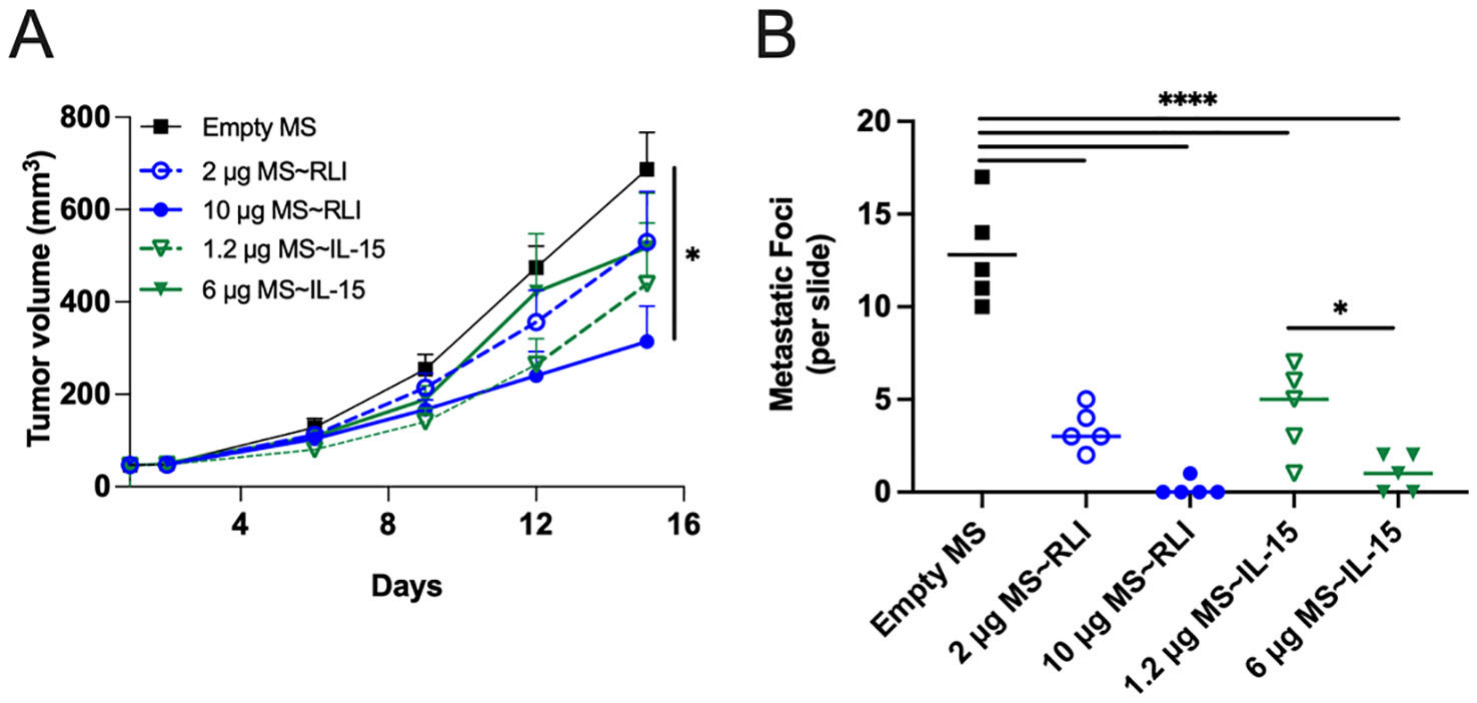
Anti-tumor effects of MS~RLI and MS~IL-15 in EO771 tumor-bearing C57BL/6 mice. Mice (n=5/group) were treated with empty MSs, MS~RLI (2 or 10 µg) or equimolar MS~IL-15 (1.2 µg or 6 µg). **(A)** Tumor growth curves. Tumor volumes are represented as mean ± SEM and statistical significance was calculated by 2 way mixed-effects analysis, *p<0.05. **(B)** Histologic analysis of metastatic lung lesions on D15 (bar indicates mean). The statistical significance was calculated by one-way ANOVA followed by Tukey’s multiple comparisons test. All MS~RLI and MS~IL-15 treated groups differ from the empty MS control (****p<0.0001). *p<0.05. Representative images from the histology slides are found in **Supplementary Figure 17**.

We studied the effects of single IT doses of MS~RLI and MS~IL-15 on the hetIL-15-sensitive EO771 (31). Mice bearing ~40 mm^3^ EO771 orthotopic tumors were treated with single IT doses of 2 or 10 µg MS~RLI or equimolar doses of 1.2 or 6 µg MS~IL-15 (**Supplementary Figure 12**). IT MS~IL-15 and MS~RLI were well tolerated with no observed body weight loss (**Supplementary Figure 13**). Treatment with 10 µg IT MS~RLI resulted in a moderate ~42% TGI and a 76% reduction in terminal tumor weight, whereas equimolar MS~IL-15 gave only ~20% TGI and insignificant ~30% tumor weight reduction (**Figure 7A**; **Supplementary Figure 14**). Despite the fact that only the high dose MS~RLI showed significant TGI, histological analysis of lung tissues showed a significant reduction in the number of metastatic lesions in mice treated with 1.2 µg MS~RLI IT or 2 µg MS~IL-15 IT (**Figure 7B**). Complete blood count of tumor-bearing animals on days -6, 2, and 14, showed in general no significant changes in circulating white blood cell populations. A ~2-fold increase in the %CD8^+^ T cells in splenocytes was observed after IT MS~RLI and IT MS~IL-15 treatment (**Supplementary Figures 15** and **16**). Taken together, these results show that IT administration of the long-acting MS~RLI/IL-15 conjugates caused moderate tumor reduction and high anti-metastatic effects.

## Discussion

We previously reported a long-acting delivery system for IL-15 that was composed of hydrogel MS tethered to IL-15 by a releasable linker, designated as MS~IL-15 (25). The aminopropyl-appended IL-15 released from SC MS~IL-15, IL-15_AP_, showed a long initial half-life of ~168 h and extraordinary effects on expansion of NK and CD44^hi^CD8^+^ T cells. The increased half-life and efficacy provided by MS~IL-15 prompted analogous studies of MS conjugates containing more potent IL-15 agonists. Herein, we describe the preparation and properties of a long-acting MS conjugate of the IL-15 agonist, RLI.

The MS~RLI conjugate was prepared by the two-step procedure used for MS~IL-15. First, an azido-linker-aldehyde was attached to the N-terminus of RLI via reductive alkylation. Then, the azido-linker-RLI was attached to cyclooctyne-activated MSs by SPAAC. The purity of RLI on the MSs was >90% and MS~RLI released RLI_AP_ with a half-life of ~700 h that maintained its functionality to bind IL-2/IL-15Rβγ_c_.

After SC administration of MS~RLI in immunocompetent mice, the released RLI_AP_ showed an apparent half-life of 30 h, some 6-fold lower than its *in vitro* half-life or the *in vivo* half-life of IL-15_AP_ released from MS~IL-15. The longer *in vivo* half-life of MS~IL-15 compared to MS~RLI is likely due to there being no consumption of the released IL-15_AP_ for 5 days, as the single chain IL-15 did not induce a “cytokine sink” as effectively as heterodimeric IL-15/IL-15Rα complex. SC administration of MS~RLI in immunodeficient mice resulted in an *in vivo* half-life of released RLI_AP_ of ~700 h, in excellent agreement with the half-life for *in vitro* release. The high release rate of MS~RLI in immune-competent mice could be closely simulated by a TMDD model, similar to that reported for the IL-15 agonist XmAb24306 (32), where the amount of target immune cells increases exponentially over time due to RLI-stimulated NK and T cell expansion – an expanding “cytokine sink”.

When administered SC, MS~RLI is ~10- and 20-fold more effective at increasing CD44^hi^CD8^+^ T cells and NK cells, respectively, than MS~IL-15 – in accord with the higher potency of RLI. These findings, combined with others, re-enforce that the heterodimeric IL-15/IL-15Rα complex has superior *in vivo* activity compared to single chain IL-15. However, after 1- to 2-weeks most mice treated with ≥20 μg SC MS~RLI showed ulcerating lesions at injection sites – histologically confirmed as severe transmural coagulative necrosis – reminiscent of the injection-site necrosis reported with a slow-release IL-2 in dextran MSs (33). The skin lesions were not seen in immunodeficient NSG mice, in agreement with the hypothesis that they were due to local immune-activation/inflammation caused by the continuous high local exposure to RLI_AP_ slowly released from the depot. Interestingly, skin reactions did not occur with free RLI injections, or with 40-fold higher doses of MS~IL-15 (25). Regardless of mechanism, the toxicity of MS~RLI occurs at a precarious 2-fold higher concentration than its most effective safe dose.

In view of the skin necrosis and low therapeutic index for SC MS~RLI, we pivoted our efforts toward studying the utility of MS~RLI for long-acting IT therapy, where some degree of necrosis might be beneficial. A significant advantage of IT administration is that low doses can achieve high local concentrations in the setting of low systemic exposure. However, the rapid escape of most locally injected therapies requires the use of multiple sequential doses for efficacy, and undermines potential advantages of IT therapy; for example, the IT half-life of a protein of ~13 kDa such as single-chain IL-15 is only ~1 h (34, 35).

Recently, it has been shown that multiple IT injections of IL-15 in MC38 colon carcinoma tumors suppressed tumor growth during the period of IL-15 administration, after which they rapidly grew (36). Also, the Pavlakis group (31) showed that multiple peri-tumoral injections of hetIL-15, the heterodimeric IL-15/IL-15Rα (7, 10) in EO771 TNBC tumors resulted in significant tumor regression, increased host survival and prevention or elimination of metastasis. We posit that instead of multiple IT injections of IL-15 agonists over time, injection of long-acting MS~RLI or MS~IL-15 conjugate would provide prolonged exposure to the continuously released cytokine, resulting in enhanced pharmacologic and immunologic responses.

The effect of single-agent IL-15 agonists on the growth inhibition of preclinical model tumors is not uniform, ranging from minimal or no to highly potent responses (30, 37–41). Likewise, single agent IL-15 agonists have not been very successful in treating human tumors, and the initial trials of single-chain IL-15 in high doses required to induce immune changes resulted in dose-limiting toxicities with no clinical response (42); the consensus is that maximal efficacy of IL-15 will require combinations with other anti-cancer agents, such as the tumor specific monoclonal antibodies CTLA-4 and PD-L1 (6, 43). Nevertheless, an important potential utility of IT IL-15 agonists is as anti-metastatic agents that can be administered in smaller local doses. Notably, IL-15^-/-^ mice injected IV with cancer cells showed high levels of metastasis, whereas IL-15^-/-^ mice treated with IL-15 showed virtually no metastasis (44). Also, it has been well established that systemically administered IL-15 agonists – single-chain IL-15 (45, 46), hetIL-15 (47), N-803 (48) and RLI (13, 49) – all have potent anti-metastatic effects, and recently it was reported that multiple peritumoral injections of hetIL-15 showed impressive anti-metastatic activity and a long-lasting specific anti-tumor immunity (31). Here, we show that a single IT injection of MS~RLI or IT MS~IL-15 resulted in moderate tumor growth inhibition but strong reduction of lung metastases. The antimetastatic effects of IL-15 agonists have been proposed to involve NK cells (49–51). Work on an orthotopic TNBC mouse model determined that systemic hetIL-15 acts both at the level of cancer cell dissemination in the blood and at the distal tissue sites to decrease detectable metastases (47). At very low SC doses, both MS~RLI and MS~IL-15 did not affect immune cells significantly. Higher doses of these IL-15 agonists administered IT showed significant anti-metastatic effects. Thus, use of IT MS~RLI or MS~IL-15 alongside with other agents could be advantageous for treating the primary tumor and preventing remote metastatic lesions.

We posit that there are multiple circumstances where IT administration of MS~IL-15 agonists in combination with systemic or local administration of other agents could provide dual benefits of anti-tumor and anti-metastatic effects, without the toxicities associated with systemic IL-15. First, therapies requiring frequent IT doses of IL-15 would be simplified to a single or very few doses. For example, the optimal efficacy of near-infrared photoimmunotherapy (NIR-PIT) requires at least four injections of IT IL-15 over 6 days to achieve the needed exposure (36). Second, local MS~IL-15 agonists would be a safe alternative for potentially toxic systemic IL-15 doses when used in combination with synergistic agents such as checkpoint inhibitors (30); indeed, the higher IT concentrations compared to systemic administration could also confer higher efficacy. Last, IT MS~IL-15 agonists could enable safe use with a second agent that has overlapping toxicities. For example, the combination of IT MS~IL-15 with synergistic agents like IL-12 (52), CAR T and NK cells (53, 54), and T cell engagers (55) could enhance antitumor activity without potential for severe, systemic overlapping toxicities such as cytokine release syndrome. To allow such combinations, MS~IL-15 agonists would be deposited into the tumor along with concurrent systemic treatment with the second drug; the tumor would be exposed to both agents whereas other tissues are exposed only to the systemically administered agent (56).

In summary, SC administration of a long-acting MS conjugate of the IL-15 agonist RLI releases RLI continuously, resulting in an apparent half-life of 30 h in immunocompetent mice – long compared to most IL-15 agonists (28), but shorter compared to the apparent half-life in NSG mice. The half-life decrease in normal mice occurs because of a rapidly expanding cytokine sink composed of lymphocytes expressing the IL-15Rβγ. The released cytokine shows very potent stimulation of NK and CD44^hi^CD8^+^ T cells but causes serious skin lesions at doses ~2-fold higher than its most effective dose. Loco-regional IT injection of MS~RLI causes expected local effects of IL-15 agonists and a strong anti-metastatic effect. Should the latter translate to humans, we propose that combinations of an effective IL-15 agonist such as MS~IL-15 with systemic or local treatment of a second agent could serve the dual purpose of improved antitumor and anti-metastatic activities achieving better efficacy, and less toxic effects compared to combinations with systemic IL-15 agonists. Importantly, the anti-metastatic effects of IT-administered long-acting IL-15 agonists overcome two of the major criticisms of loco-regional therapy: the need for frequent injections, and the complexity of managing metastasis (57, 58).

## Data Availability

The original contributions presented in the study are included in the article/**Supplementary Material**. Further inquiries can be directed to the corresponding author.
