# Supplementary material for: Leveraging long-acting IL-15 agonists for intratumoral delivery and enhanced antimetastatic activity
Source: Front Immunol. 2024 Nov 4;15:1458145. doi: 10.3389/fimmu.2024.1458145 (PMC11570272; doi:10.3389/fimmu.2024.1458145)
Supplement: Supplementary file 1 [file DataSheet1.pdf]

Supporting Information

Leveraging long-acting IL-15 agonists for intratumoral delivery and enhanced antimetastatic activity

John A. Hangasky[1†], Rocio del Valle Fernandez[1†], Dimitris Stellas[2], Guillermo Hails[1], Sevasti Karaliota[2,3], Gary W. Ashley[1], Barbara K. Felber[2], George N. Pavlakis[4], and Daniel V. Santi[1*]

[1]ProLynx Inc., San Francisco, CA 94107, USA
[2]Human Retrovirus Pathogenesis Section, Vaccine Branch, Center for Cancer Research, National Cancer Institute at Frederick, Frederick, MD 21702, USA
[3]Basic Science Program, Frederick National Laboratory for Cancer Research, Leidos Biomedical Research, Inc., Frederick, MD 21702, USA
[4]Center for Cancer Research, National Cancer Institute at Frederick, Frederick, MD 21702, USA

† These authors contributed equally
* Corresponding author: Daniel V. Santi, ProLynx Inc, 135 Mississippi St, San Francisco, CA 94107; Daniel.V.Santi@prolynxinc.com. Phone 415 665-5775

Table of Contents

I. Analytical procedures for RLI pg. 2
II. Expression and characterization of RLI pg. 4
III. Preparation of MS~RLI conjugates pg. 5
IV. Characterization of MS~RLI pg. 6
V. In vivo studies pg. 8
VI. Therapeutic Studies pg 10
VII. Supplemental References pg.16

Figures and Tables

Table S1. Antibodies used for immunophenotyping
Fig. S1. Representative gating strategy.
Fig. S2. Deglycosylation of RLI with PNGase F.
Fig. S3. Reductive alkylation of RLI.
Fig. S4. In vitro release kinetics
Fig. S5. Purity of RLI bound to the microspheres.
Fig. S6. Bioactivity of RLI_{AP}.
Fig. S7. MS~RLI elicits expansion of target immune cells.
Fig. S8. Body weights of naïve C57BL/6J mice following treatment
Fig. S9. PD response following two doses $MS\text{~}RLI_{10µg.}$
Fig. S10. Body weights of CT26 tumor bearing mice following treatment
Fig. S11. Immunophenotyping of immune cells in CT26 tumor bearing mice 5 days after treatment.
Fig. S12. Treatment schedule for EO771 tumor bearing mice.
Fig. S13. Body weights of EO771 tumor bearing mice following treatment.
Fig. S14. Final weight of EO771 tumors.
Fig. S15. Flow cytometric analysis of immune cells in tumors & spleen in EO771 tumor bearing mice.
Fig. S16. Complete blood count analysis following IT MS~RLI or MS~IL-15.
Fig. S17. H&E staining of lungs.

I. Analytical procedures for RLI

HPLC analysis.

HPLC analyses were performed on a Shimadzu LC-20AD HPLC system equipped with a Phenomenex Jupiter 5 μM C18 column (300 Å, 150 x 4.6 mm) heated to 40°C and a SPD-M20A photodiode-array detector. The elution program consisted of a 10-min linear gradient from 20- to 100% CH_3CN containing 0.1% TFA with a flow rate of 1 mL/min.

U2OS Dimerization Cell Based Assay.

A U2OS cell-based assay kit for IL-2Rβγ binding was performed according to the manufacturer's instructions (DiscoverX, Part #93-0998E3CP5). Briefly, cells were plated (100 μL, ~5,000 cells/well) in 96 well white-walled assay plates and incubated for 48 hours at 37ºC, 5% CO_2. Cells were treated with varying concentrations of the RLI or RLI_{AP} and incubated for an additional 6 hours at 37ºC, 5% CO_2. Treated cells were then incubated with the PathHunter reagent substrate for 1 hour at ambient temperature protected from light. Plates were read for chemiluminescence signal detection using a Spectramax i3 plate reader using a 250 ms integration time.

ELISA.

The RLI concentrations in plasma were assessed using either a hIL-15/IL-15Rα complex specific ELISA (R&D Systems, hIL-15/IL-15Rα complex DuoSet ELISA, Catalog #DY6924) or ELLA protein simple kit (Catalog SPCKB-PS-000500) performed according to the manufacturer's instructions. Plasma samples were thawed on ice prior to 4- to 10-fold dilution in the provided standard diluent. RLI concentrations were plot as a function of time and fit using GraphPad Prism software.

Cell staining protocols

Pharmacodynamic studies in naïve mice. EDTA whole blood (25 μL) was transferred to a 96 deep well plate and incubated with a fixable viability dye to label dead cells. FcRγII/II receptors were blocked with CD16/32 (20 μL, 1.6 μg) before staining for cell-surface antigens (all reagents from Invitrogen). The whole blood samples were incubated at 4°C for 30 minutes with previously determined optimal antibodies concentrations for surface staining of peripheral blood mononuclear cells (PBMCs) (**Table S1**). Red blood cells were lysed and PBMCs were fixed by incubation with 2 mL of 1-Step Fix/Lyse solution for 30 minutes at room temperature. Fixed cells were washed once with 2 mL permeabilization buffer and then intracellularly stained (Ki-67-APC) using a 30 minutes incubation period. After the cells were stained, samples were washed 2X with

2 mL of FACS buffer. The cells were resuspended in 400 µL FACS buffer and stored at 4°C until analysis.

Tissue immunophenotyping in CT26 tumor bearing mice. Single cell suspensions of splenocytes were obtained from harvested spleens following mechanical disruption and filtering through a 40-µm cell strainer. RBCs were lysed using 1X RBC Lysis Buffer (Invitrogen). Tumor infiltrating lymphocytes (TILs) were obtained by excision and dissociation of tumor tissue. Tumors were minced into small pieces and treated with 3 mg/mL of Collagenase 4 (ThermoFisher) and 0.5 mg/mL of DNase I (Roche) for 30 min. at 37ºC. Then, the dissociated tissue was filtered through a 40-µm cell strainer and cell counts were determined using a Via1 Cassette (ChemoMetec). Single cell suspensions (<$2x10^7$cells/ml) were prepared and lymphocytes were purified by centrifugation on a Lympholyte-Mammal Cell Separation Media gradient (Cedarlane) for 20 min at 1250 g, 20ºC with slow acceleration and brakes turned off.

Aliquots of these cell suspensions (80 µL; ~$1x10^6$ cells), as well as aliquots of blood (50 µL), were stained for FACS analysis. Briefly, a fixable viability dye was used to label dead cells (LIVE/DEAD™ Fixable Aqua Dead Cell Stain Kit, Invitrogen), followed by an incubation with CD16/32 antibody to block FcRγII/II. Then samples were surface stained with previously determined optimized Ab concentrations for three different panels (all reagents from eBioscience, **Table S1**). RBCs from blood samples, were lysed with 1x RBC lysis buffer (Invitrogen). Intracellular marker staining was performed using the Foxp3 Transcription Factor Staining Buffer Set (Invitrogen) and following supplier instructions.

Stained single cell suspensions were read using a Attune NxT flow cytometer (BD Biosciences) and analyzed using FlowJo cytometry analysis software (TreeStar, Ashland, OR). The absolute cell numbers of the samples were determined by direct cell analysis (volumetric counting by the Attune NxT). Positive populations were identified based on fluorescence minus one control.

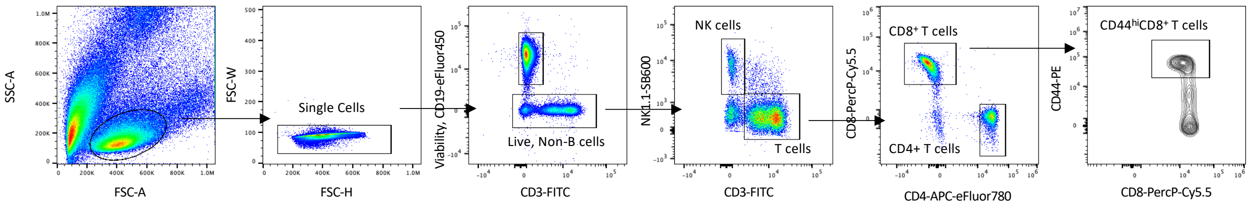

Figure S1. Representative gating strategy.

Table S1. Antibodies used for immunophenotyping

Antibody	Clone	Vendor
αCD45-APC-eFluor780	30-F11	Invitrogen
αCD3e-FITC	145-2C11, 17A2	Invitrogen
αCD8a-PerCP-Cy5.5	53-6.7	Invitrogen
αCD4-eFluor780	GK1.5	Invitrogen
αCD4- PerCP-Cy5.5	RM4-5	Invitrogen
αNK1.1-SuperBright600	PK136	Invitrogen
αNKp46-PE	29A1.4	Invitrogen
αCD49b-PerCP-eFluor710	DX5	Invitrogen
αCD44-PE	IM7	Invitrogen
αKi67-APC	SolA15	Invitrogen
αCD19-eFluor450	1D3	Invitrogen
αCD19-eFluor506	1D3	Invitrogen
αCD25-PE	PC61.5	Invitrogen
αFoxp3-eFluor450	FJK-16s	Invitrogen
Live/Dead Fixable Aqua dead cell stain	—	Invitrogen
αCD16/CD32	2.4G2	BD Pharmingen

II. Expression and characterization of RLI

RLI (>95% pure, molecular weight: 22841 Da) was produced at ATUM (Newark, CA) based on previously reported methods (1). The purified protein was observed as distinct bands by SDS-PAGE and eluted as two peaks in HPLC chromatograms. When treated with PNGase F deglycosylase and evaluated by SDS-PAGE, conversion of the upper band into the lower band was observed indicating the higher molecular weight band is glycosylated RLI (**Fig. S2**).

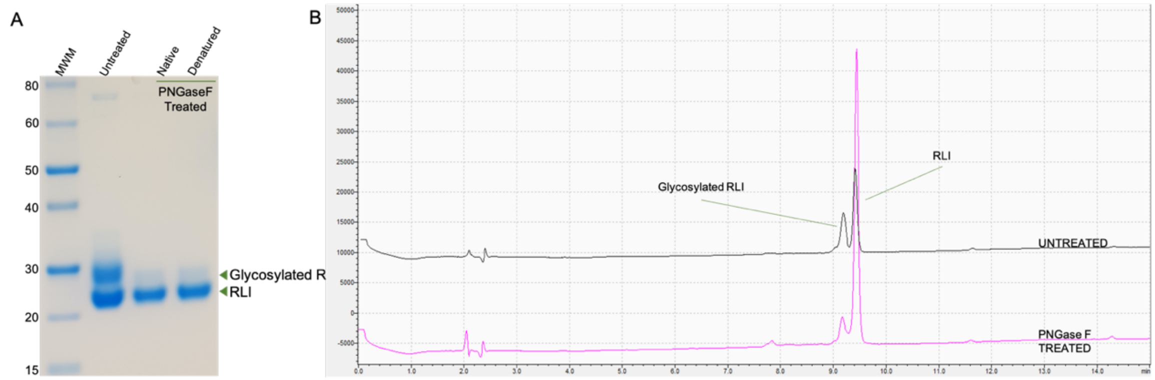

Figure S2. Deglycosylation of RLI with PNGase F. A) SDS-PAGE analysis of RLI. Lane 1: Molecular weight marker; Lane 2) Untreated RLI sample; Lane 3: RLI treated with PNGase F; Lane 4: heat denatured RLI was treated with PNGase F. B) HPLC chromatogram of RLI before

and after treatment with PNGaseF. Native RLI or heat denatured RLI was treated with PNGase F (0.4 mU/μg RLI) at 37°C for 48 hours.

III. Preparation of MS~RLI conjugates

Optimization of azido-linker-RLI yield. Small scale reductive alkylation reactions of RLI varying the linker concentration were performed to determine optimal reaction conditions for stoichiometric linker addition to RLI. Reactions (50 μL) used 10 nmol (0.2 mg, 200 μM) RLI in 25 mM Citrate, pH 6.0, 500 mM NaCl and 0.05% tween-20 (Buffer A), 1.5- to 5 equivalents of N_3-PEG_4-L($MeSO_2$)-CHO (9 μg to 29 μg, 300 μM to 1 mM) and 750 nmol (47 μg, 10 mM) $NaCNBH_3$. After 20 hours at room temperature, mixtures were treated with 0.5 mM DBCO-PEG_{5kDa} for 4 hours, and analyzed by SDS-PAGE (**Fig. S3**) (2).

A

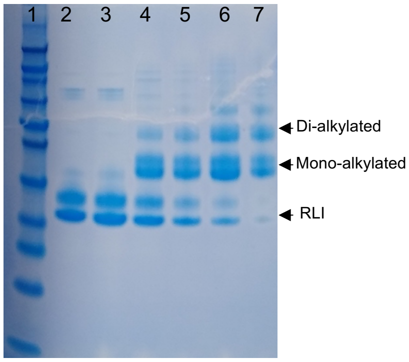

B

Linker eq	Unmodified	Mono-alkylated	Di-alkylated	Tri-alkylated
1.5	46%	44%	10%	–
2.0	33%	53%	14%	–
3.0	14%	53%	27%	6%
5.0	5%	48%	28%	19%

Figure S3. Reductive alkylation of RLI. **A)** SDS-PAGE of RLI performed with varying amounts of N_3-PEG_4-L($MeSO_2$)-CHO showing free-, mono- and multi-alkylated RLI. Alkylated RLI was reacted with DBCO-PEG_{5K} to slow migration of alkylated RLI species. L1, Novex Sharp Pre-Stained Protein Ladder; L2, RLI; L3, RLI + PEG_{5KDa}; L4, N_3-linker:RLI_{RM} = 1.5; L5, N_3-linker:RLI_{RM} = 2.0; L6, N_3-linker:RLI_{RM} = 3.0; L7, N_3-linker:RLI_{RM} = 5.0. **B)** The percent of RLI modified, as determined from the SDS PAGE gel. Bands were quantified using ImageJ software. RLI (10 nmol) was treated with 1.5 – 5 equivalents N_3-linker-CHO (Mod = $MeSO_2$) and $NaCNBH_3$ (10 mM) in 25 mM Citrate 500 mM NaCl and 0.05% tween-20 for 20 hours at room temperature in the dark.

Preparation of azido-linker-RLI. In 4 mL of Buffer A, reaction mixtures contained 0.8 μmol (18 mg, 200 μM) RLI, 1.6 μmol (0.9 mg, 400 μM) of N_3-PEG_4-L($MeSO_2$)-CHO and 40 μmol $NaCNBH_3$ (10 mM). The reaction was allowed to proceed 20 hours at ambient temperature protected from light. The excess reagents were removed using two 14.5 x 50 mm PD-10 columns (GE Healthcare) previously equilibrated in Buffer A. A small aliquot of the reaction mixture was reacted with DBCO-PEG_{5kDa} for 4 hours and analyzed by SDS-PAGE. The gel-shift assay indicated the sample contained ~39% unreacted-, 51% monoalkylated- and 10% dialkylated-RLI. The reaction mixture was concentrated to ~0.8 mL using an Amicon Ultra 10,000 MW cut-off concentrator and the

protein concentration (17 mg, 94% recovery) was determined by A_{280} (ε_{280}= 22,960 M^{-1} cm^{1}) using a NanoDrop spectrophotometer.

Preparation of MS~RLI. A slurry of 0.51 mL of BCN-derivatized MSs containing 1.6 µmol BCN/mL in a 10 mL sterile syringe was washed with 5 x 7 mL of Buffer A. The azido-linker-RLI reaction mixture containing 0.74 µmol (17 mg) of ~50% mono-alkylated RLI in 1.5 mL Buffer A was added to the syringe through a 0.22 µm sterile filter. The mixture was rotated end-over end at ambient temperature for 48 hours, and washed with 5 x 6 mL of Buffer A to remove unbound RLI. The unreacted BCN groups were capped by treatment with 0.1 mL of 50 µmol/mL N_3-PEG_7 (Sigma Aldrich) for 24 hours, then MSs with 6 x 7 mL of Buffer A containing 30 mM Met and stored at 4°C. Protein content of the microspheres was determined by A_{280} in 10 mg aliquots of slurry after dissolution in 40 µL of 50 mM NaOH (ε_{280}= 22,960 M^{-1} cm^{1}). The PEG content of the conjugate was determined following dissolution using $BaCl_2/I_2$ spectrophotometry.

IV. Characterization of MS~RLI

In vitro release kinetics and reverse gelation time of MS~RLI. The release kinetics and reverse gelation time of MS~RLI were determined under accelerated release conditions. The MS~RLI slurry was washed twice using a 25-fold dilution in 125 mM borate buffer pH 9.4. After the second wash, MS~RLI was diluted 10-fold in 125 mM sodium borate pH 9.4, and incubated at 37°C. At predefined time intervals, the reaction mixture was centrifuged and the supernatant was sampled. A Nanodrop spectrophotometer was used to determine the A_{280} of supernatant (ε_{280}= 22,960 M^{-1} cm^{1}). Each sample was also analyzed by HPLC. The release rate was calculated by fitting the released A_{280} vs time or RLI peak area vs time to a first-order rate equation in GraphPad Prism 8.0, and the $t_{1/2}$ of RLI release at pH 7.4 was calculated using the equation:

$$t_{1/2,\ pH\ 7.4} = t_{1/2,\ pH} \times 10^{(pH-7.4)}$$

The in vitro solubilization of the MSs was monitored by quantitating the amount of PEG in the supernatant by $BaCl_2/I_2$ spectrophotometry. Time matched samples (5 µL) of the reaction supernatant from the in vitro release assay were diluted 20-fold in water. The diluted sample (100 µL) was acidified with perchloric acid (0.5 M, 500 µL). In a 96-well plate, the acidified sample (200 µL) was treated with a solution (75 µL) of $BaCl_2$ (3.3% w/v), I_2 (0.06%) and KI (0.12%) and the absorbance was measured at 535 nm. The amount of PEG in the solution was calculated based on a linear standard curve generated by the same procedure using a solution of linear PEG_{8KDa} (7.5 – 60 µg/mL).

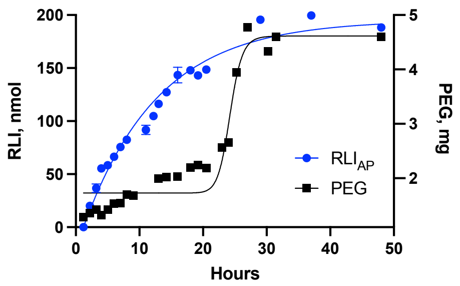

Figure S4. In vitro release kinetics. The release of RLI$_{AP}$ (●, $t_{1/2,pH9.4}$= 7 h) and degelation (■, $t_{RG,pH\ 9.4}$= 28 h) of MS~RLI**.**

Purity of RLI on MS~RLI. Purity of the RLI conjugated to the microsphere was determined by monitoring the released proteins at pH 9.4, 37ºC by HPLC. The MS~RLI slurry was washed two times and diluted in 125 mM sodium borate pH 9.4, and incubated at 37°C. At intervals spanning over 8 h , (~1 half-life of release) samples were centrifuged and 20 µL of the supernatants were analyzed by HPLC. The fraction of each released protein was determined as peak area/total peak areas, was plotted. The y-intercept of the plot reveals the fraction of each protein present on the MSs at t=0.

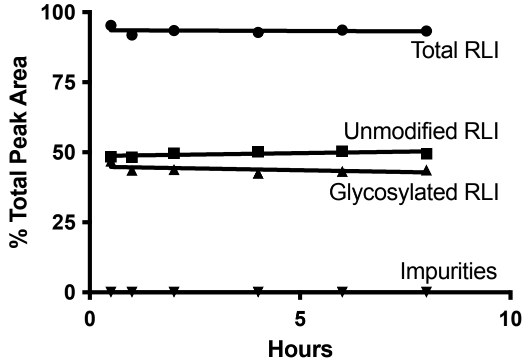

Figure S5. Purity of RLI bound to the microspheres. Released proteins from the microspheres monitored by HPLC. The analysis shows >93% of the protein on the MSs is RLI. Approximately 45% of the RLI loaded to the MSs is glycosylated.

Bioactivity of RLI$_{AP}$. In a 1.5 mL microcentrifuge tube, 50 µL of the MS~RLI conjugate (0.2 mg, 8.7 nmol) was washed 1x with PBS pH 7.4 and then diluted 10 fold in the same buffer. To obtained RLI$_{AP}$, the washed microsphere conjugate was incubated in a 37ºC water bath for 10 days. The supernatant of the reaction mixture, containing RLI$_{AP}$, was concentrated to 0.18 mg/mL (7.8 µM) using an Amicon Ultra 3,500 MW cut-off concentrator. The in vitro activity of RLI$_{AP}$ compared to RLI was assessed using the U2OS cell-based assay kit for IL-2Rβγ binding.

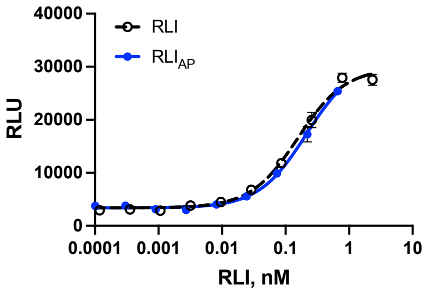

Figure S6. Bioactivity of RLI_{AP}. RLI (○) and RLI_{AP} (●) were assayed for IL-2/IL-15Rβγ binding via an U2OS cell-based assay. The EC_{50} for RLI induced dimerization of IL-2/IL-15Rβγ by RLI (EC_{50} = 160 pM) and RLI_{AP} (EC_{50} = 200 pM) was determined by fitting data to a four-parameter logistic model; data points represent mean ± SD.

V. In vivo studies

Preparation of dosing solutions. Dosing solutions were prepared by diluting the MS~RLI slurry in 25 mM Na citrate buffer pH 5.9 containing 500 mM NaCl, 0.05% tween-20 and 1.25% (w/v) hyaluronic acid. The RLI concentration was confirmed by diluting aliquots of the MS~RLI dosing solution (~20 µL) in 50 mM NaOH (80 µL) and incubated for 1 hour at room temperature. Following a 1 hour room temperature incubation, the reaction mixture (20 µL) was analyzed using standard HPLC methods. A standard curve was prepared using native RLI standards (0.047 – 0.856 mg/mL) prepared from 2-fold serial dilutions in 50 mM NaOH. The concentration of RLI in each dosing solution was determined by peak area interpolation of the standard curve. Following the same protocol, the IL-15 content content of MS~IL-15 dosing solutions were quantitated by HPLC using IL-15 standards (0.004 – 0.25 mg/mL). Equimolar doses of MS~RLI and MS~IL-15 were determined using molecular weight of RLI (22841 Da) and IL-15 (12901 Da).

Pharmacokinetics of MS~RLI. Syringes with fixed needles (27 G) were backfilled with the MS~RLI conjugate (100 µL). The contents of the syringes were administered SC to either male C57BL/6J mice or male NSG mice 6-8 weeks old. For studies in C57BL/6J mice, blood samples were collected in EDTA collection tubes at -48, 8, 24, 48, 96, 120, 168 and 240 hours from alternating groups of mice (n=3/group). For studies in NSG mice, blood samples were collected in EDTA collection tubes at -48, 8, 24, 48, 72, 96, 120, 168, 240, 288, 336, 360, 408,456, 504, 552, 624 and 672 hours from alternating groups of mice (n=3/group). HALT protease inhibitor cocktail was added to all samples and the plasma frozen at –80°C until analysis by ELISA.

Pharmacodynamic Studies

Naïve, male C57BL/6J mice (n=4-5/group) 6-8 weeks old were administered SC MS~RLI (1 – 10 µg) or MS~IL-15$_{50µg}$ in the mid-back or a single IP dose of free RLI (10 µg), or two IP doses of 2 µg RLI (D0 and D2). Mice that were administered sequential doses of MS~RLI or free RLI, were administered a cycle of treatment starting on Day 35. Blood samples were drawn on day -2, 2, 5, 7, 14, 21 and 28. For mice that received multiple doses, additional blood samples were collected 2, 5, 7, 14, and 21 days post second injection. PBMCs were prepared and immunophenotyped with an Attune NxT flow cytometer using an 8 color T cell panel to quantitate B cells, NK cells, CD3, CD4, CD8, and CD44 expressing cells. The total AUC for each cell phenotype was determined using Prism. Then, the baseline cell count x 28 was subtracted to yield the AUC$_{28d}$.

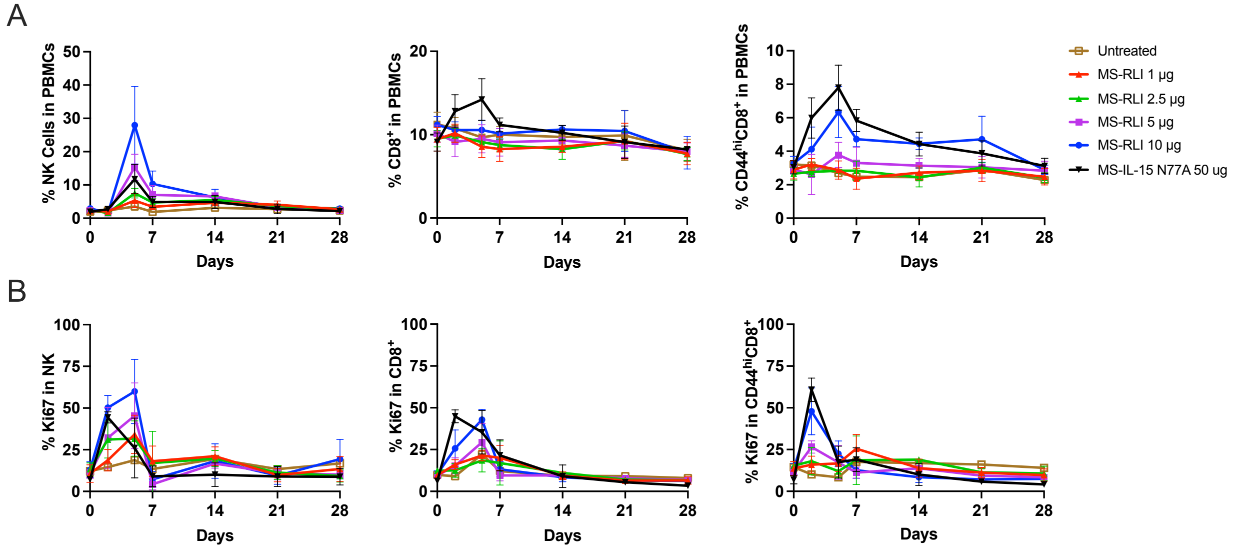

Figure S7. MS~RLI elicits expansion of target immune cells. A) Frequency of NK, CD8^{+}, and CD44^{hi}CD8^{+} T cell expansion in PBMCs. B) Percentage of proliferating NK, CD8^{+}, and CD44^{hi}CD8^{+} T cells. Mice were administered a single SC dose of MS~RLI (1 – 10 µg) or MS~IL-15 (50 µg) . Data represented as mean ± SD (n=5/group).

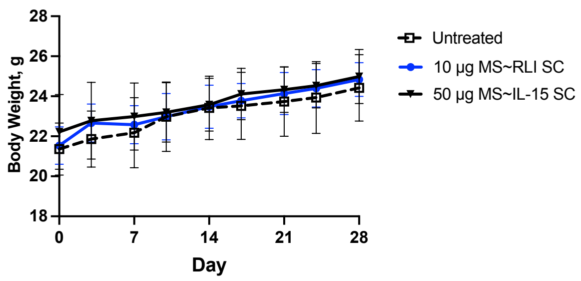

Figure S8. Body weights of naïve C57BL/6J mice. Mice were administered a single SC dose 10 µg MS~RLI (●) or 50 µg MS~IL-15 (▼). Data represented as mean ± SD (n=5/group).

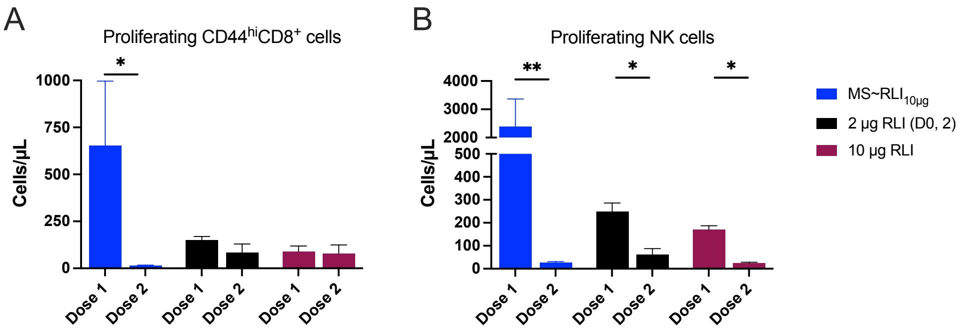

Figure S9. PD response following two doses MS~RLI$_{10µg}$. A) Absolute cell number of proliferating immune cells 5 days after dosing on D 0 (Dose 1) and D 35 (Dose 2). Mice (n=4-5/group) were administered MS~RLI$_{10µg}$ (blue), RLI$_{10µg}$ (brown) or two doses of 2 µg RLI separated by 48h (black). After 35 days, mice were treated again. Data represented as mean ± SD The statistical significance was calculated by one-way ANOVA followed by Tukey's multiple comparison test. *p<0.05, **p<0.01.

Skin toxicity of MS~RLI

On Day 0, C57BL/6J or NSG mice were weighed, anesthetized with isoflurane, and their backs were shaved. MS~RLI (10 – 30 µg, 0.43 – 1.3 nmol) was injected SC into the flank of each mouse (n=6-15/group); a control group (n=5) received a single SC injection of RLI (20 µg, 0.9) in the flank. The location of each injection was marked with a sharpie and reapplied as needed to monitor the site over 28 days. If an injection site lesion formed, the injection site was photographed and the mouse euthanized. All surviving mice were sacrificed 28 days after injection.

VI. Therapeutic Studies

CT26 syngeneic model

CT26 tumors were established in the flank of female BALB/c mice by injection of 1 x10^5 in 100 µL of serum-free medium. When the tumor volume reached ~100mm^3 mice were randomized into groups (n=7-8/group) and mice were administered empty MSs IT, 10 µg MS~RLI IT, or empty MSs IT plus 10 µg MS~RLI SC. The experiment was performed two times. In the second experiment, half of the mice (n=4/group) were sacrificed on day 5, and EDTA whole blood, tumors and spleens were harvested for immunophenotyping; tumor volumes and survival of the remaining mice (n=4/group) were recorded through day 30. The tumor volume was measured by calipers and calculated by the equation: V = 1/2(long dimension)(short dimension)^2. Kaplan-Meier mouse survival plots were generated based on the mouse survival, monitored based on humane end point criteria.

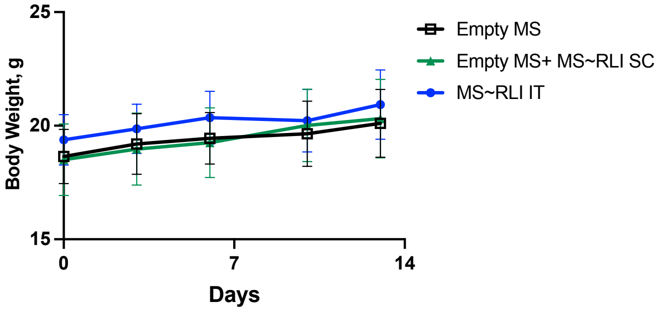

Figure S10. Body weights of CT26 tumor bearing mice following treatment. Mice were administered empty MSs IT (□), 10 μg MS~RLI IT (●), or empty MSs IT plus 10 μg MS~RLI SC (▲) (n=7-8/group).

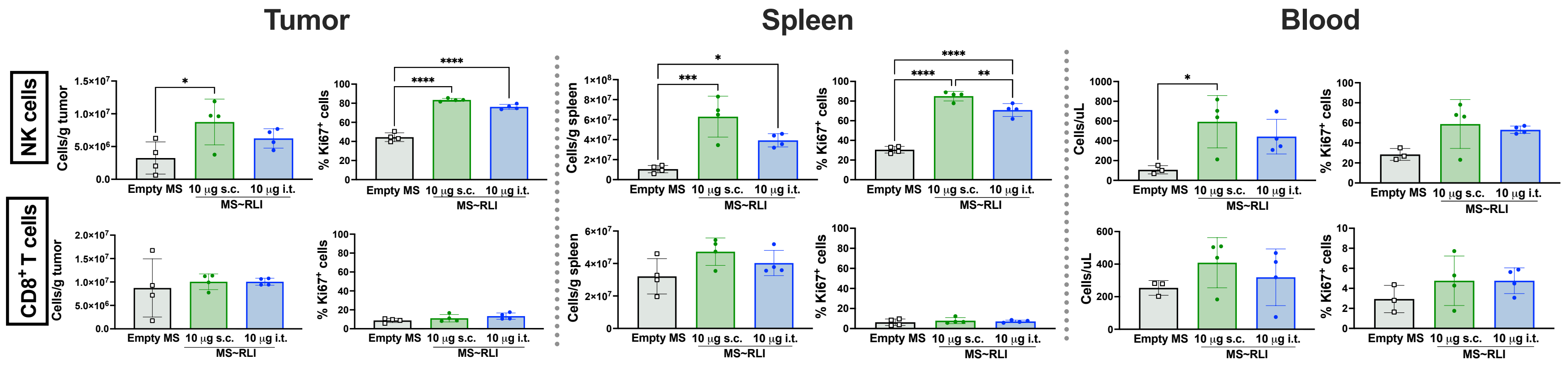

Figure S11. Immunophenotyping of tumors, spleens and blood of CT26 tumor bearing mice on D5 following treatment with 10 µg MS~RLI administered SC (●),or IT (●). Empty MS administered IT were used as untreated control (□). Graphs show the number of NK cells and CD8⁺T cells normalized to grams of tissue (spleen or tumor) or µL of blood and the percentage of proliferative cells (Ki67⁺) of every cell subtype. Results are expressed as mean ± SD. Statistical significance was determined by one-way ANOVA followed by Tukey's multiple comparison test. *p<0.05; **p<0.01; ****p<0.0001. n=3-4/group.

Orthotopic *EO771 tumor bearing mice*

EO771 cells (3x10^5 cells) were orthotopically inoculated into the 4th mammary pad of C57BL/6 mice. When the tumors reached ~40 mm^3 in size, a single dose of MS~IL-15 (1.2 µg or 6 µg) or MS~RLI (2 µg or 10 µg) were administered intratumorally in a volume of 50 µL (n=5 mice per group). Empty microspheres (50 µL) were used as the negative control and were administered intratumorally (n=5/group). Blood was collected at 3 timepoints as follows: 6 days before treatment, one day after the first injection and at the end of experiment (**Fig. S10**). Tumor volume (mm^3) was measured by calipers and calculated by the following equation: L*W*H*p/6. All mice were sacrificed on day 15 of the treatment. Tumor-infiltrating immune cells and splenocytes were analyzed by flow cytometry. Lungs were embedded in paraffin and the metastatic lesions were evaluated by hematoxylin/eosin staining.

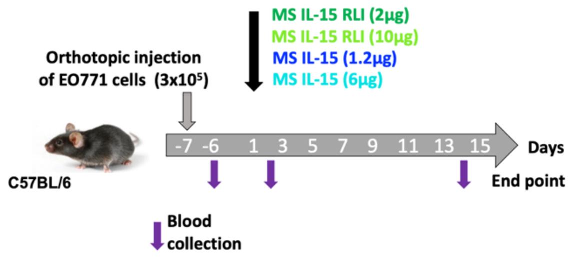

Figure S12. Treatment schedule for EO771 tumor bearing mice. On day -7, C57BL/6 mice were inoculated with 3x10^5 EO771 cells orthotopically in the fourth mammary pad. Mice with palpable tumors were distributed in different groups 7 days later and treated with MS~IL-15, MS~RLI or empty microspheres.

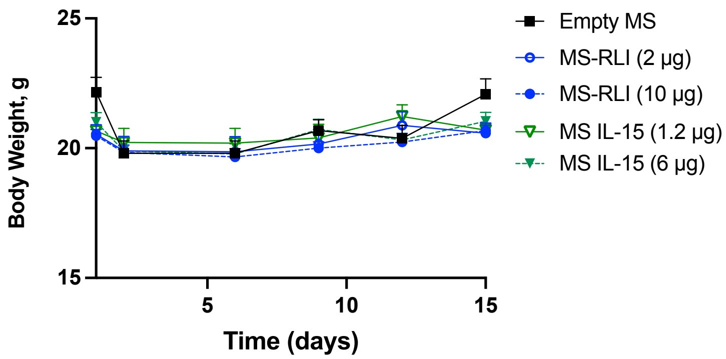

Figure S13. Body weights of EO771 tumor bearing mice following treatment. Mice were treated IT with empty microspheres (■), 1.2 µg MS~IL-15 (○), 6 µg MS~IL-15(●), 2 µg MS~RLI (▽) or 10 µg MS~RLI (▼) (n=5/group).

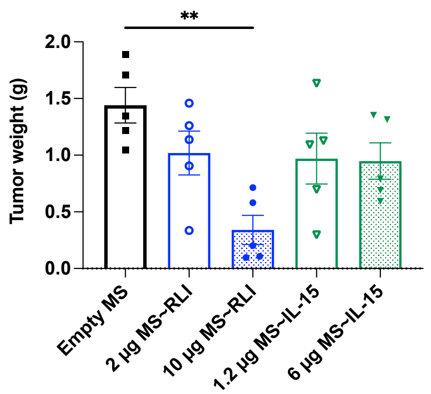

Figure S14. Final weight of EO771 tumors.

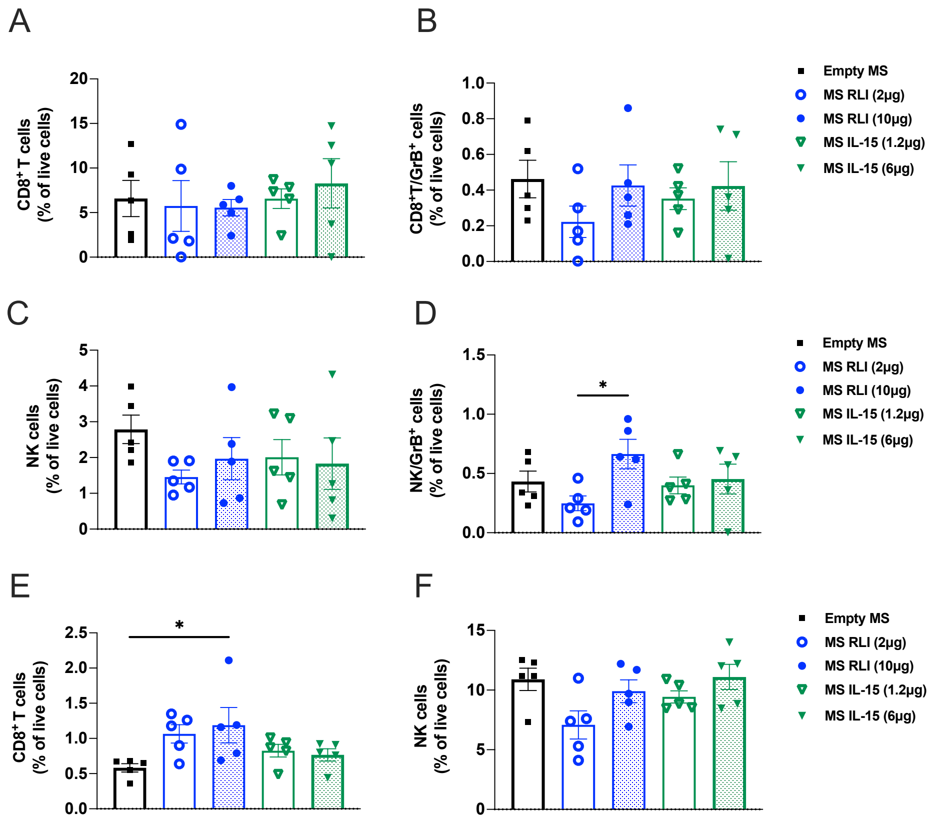

Figure S15. Flow cytometric analysis of immune cells in tumors and spleen in EO771 tumor bearing mice. A) CD8^{+} T cells, B) CD8^{+}GrB^{+} T cells, C) NK and D) NK GrB^{+} cells in tumors, and E) CD8^{+} T cells and F) NK cells in the spleen on day 15. Data in graphs are given as percentage of total live cells and represented as mean ± SEM. Statistical significance was calculated by one-way ANOVA followed by Tukey's multiple comparision test. *p<0.05

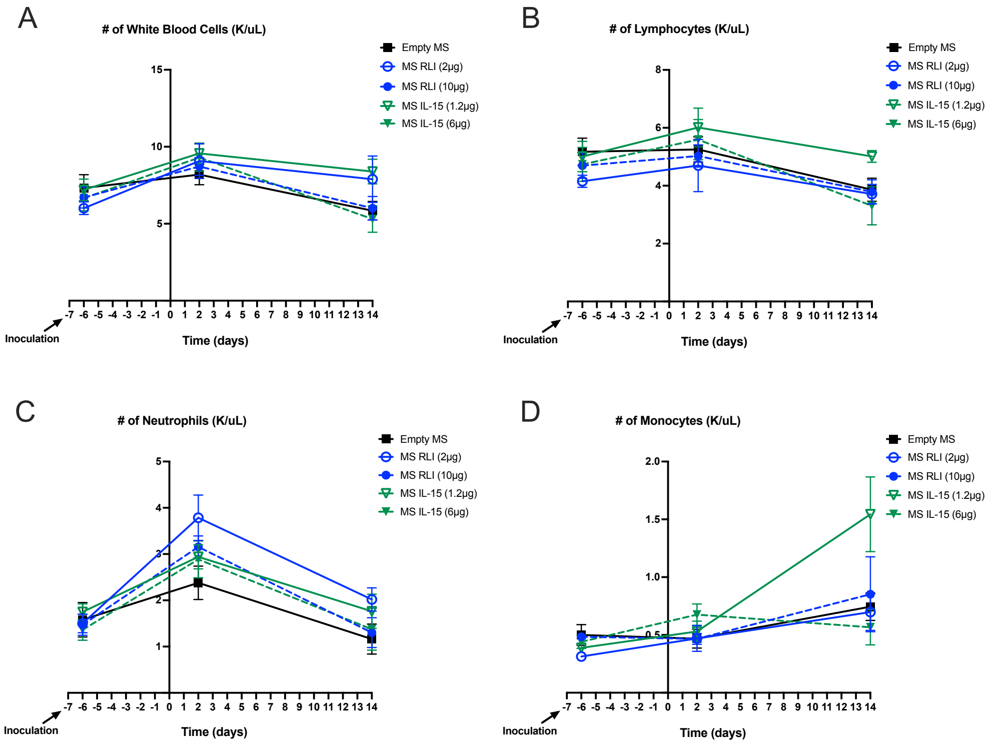

Figure S16. Complete blood count analysis following IT MS~RLI or MS~IL-15.The total number of whole white blood cells (A), lymphocytes (B), neutrophiles (C) and monocytes (D) are shown.

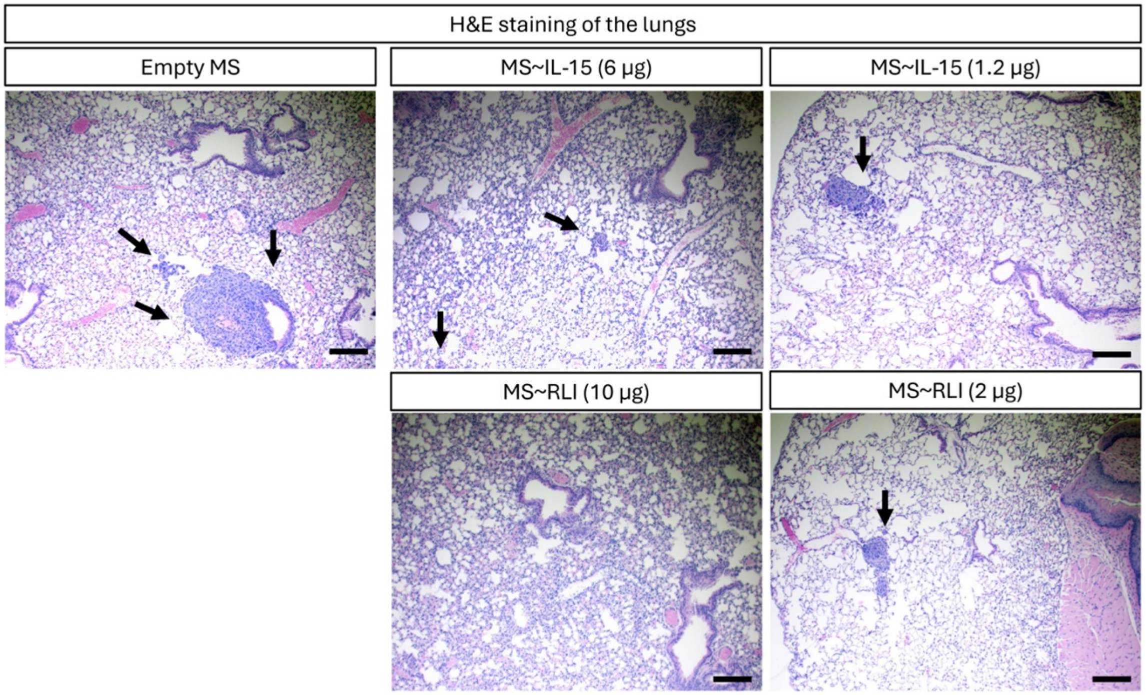

Figure S17. H&E staining of lungs. Representative images from H&E analysis of metastatic lesions in lungs of the treated and control animals. The arrows indicate the metastatic lesions; scale bar = 1000 µm.

VII. Supplemental References

1. E. Mortier, A. Quemener, P. Vusio, I. Lorenzen, Y. Boublik, J. Grotzinger, A. Plet and Y. Jacques: Soluble interleukin-15 receptor alpha (IL-15R alpha)-sushi as a selective and potent agonist of IL-15 action through IL-15R beta/gamma. Hyperagonist IL-15 x IL-15R alpha fusion proteins. *J Biol Chem*, 281(3), 1612-9 (2006) doi:10.1074/jbc.M508624200

2. E. L. Schneider, B. R. Hearn, S. J. Pfaff, S. D. Fontaine, R. Reid, G. W. Ashley, S. Grabulovski, V. Strassberger, L. Vogt, T. Jung and D. V. Santi: Approach for Half-Life Extension of Small Antibody Fragments That Does Not Affect Tissue Uptake. *Bioconjug Chem*, 27(10), 2534-2539 (2016) doi:10.1021/acs.bioconjchem.6b00469
